# Laparoscopic versus open colectomy for colon cancer in an older population: a cohort study

**DOI:** 10.1186/1477-7819-10-31

**Published:** 2012-02-07

**Authors:** Linda C Cummings, Conor P Delaney, Gregory S Cooper

**Affiliations:** 1 Division of Gastroenterology and Liver Disease, Department of Medicine, University Hospitals Case Medical Center, 11100 Euclid Avenue Mailstop 5066, Cleveland, Ohio 44106-5066, USA; 2 Division of Colorectal Surgery, University Hospitals Case Medical Center, 11100 Euclid Avenue Mailstop 5047, Cleveland, Ohio 44106-5047, USA

## Abstract

**Background:**

Laparoscopic colectomy for colon cancer has been compared with open colectomy in randomized controlled trials, but these studies may not be generalizable because of strict enrollment and exclusion criteria which may explicitly or inadvertently exclude older individuals due to associated comorbidities. Previous studies of older patients undergoing laparoscopic colectomy have generally focused on short-term outcomes. The goals of this cohort study were to identify predictors of laparoscopic colectomy in an older population in the United States and to compare short-term and long-term outcomes.

**Methods:**

Patients aged 65 years or older with incident colorectal cancer diagnosed 1996-2002 who underwent colectomy within 6 months of cancer diagnosis were identified from the linked Surveillance, Epidemiology, and End Results-Medicare database. Laparoscopic and open colectomy patients were compared with respect to length of stay, blood transfusion requirements, intensive care unit monitoring, complications, 30-day mortality, and long-term survival. We adjusted for potential selection bias in surgical approach with propensity score matching.

**Results:**

Laparoscopic colectomy cases were associated with left-sided tumors; areas with higher population density, income, and education level; areas in the western United States; and National Cancer Institute-designated cancer centers. Laparoscopic colectomy cases had shorter length of stay and less intensive care unit monitoring. Although laparoscopic colectomy patients (n = 424) had fewer complications (21.5% versus 26.3%), lower 30-day mortality (3.3% versus 5.8%), and longer median survival (6.6 versus 4.8 years) compared with open colectomy patients (n = 27,012), after propensity score matching these differences disappeared.

**Conclusions:**

In this older population, laparoscopic colectomy practice patterns were associated with factors which likely correlate with tertiary referral centers. Although short-term and long-term survival are comparable, laparoscopic colectomy offers shorter hospitalizations and less intensive care.

## Background

Over the past decade, laparoscopic and laparoscopically-assisted resection of colon cancer have emerged as promising alternatives to open colectomy. Benefits of laparoscopic colectomy (LC) over open colectomy (OC) include decreased operative blood loss, shorter hospitalization, and improved pain control [1-4]. LC is selectively practiced with T4 lesions, obstruction, or perforation, and may be challenging due to adhesions or morbid obesity. New staging information or technical difficulties arising during laparoscopy may lead to conversion to open colectomy, with rates of 5%-21% [2,5,6].

Randomized controlled trials (RCT) have shown no long-term survival difference between LC and OC [5,7-9] or, in one single-center trial, better cancer-related survival with LC [10]. However, these studies may not be generalizable because of strict inclusion or exclusion criteria which may explicitly or inadvertently exclude older individuals due to associated comorbidities. Individuals aged ≥ 65 years nevertheless have the highest incidence of colorectal cancer [11]. A recent study applying inclusion and exclusion criteria from two of these RCTs to a prospective colectomy database found that excluded patients were older [12]. Whilst older patients might benefit most from the less invasive approach offered by laparoscopic colectomy because of limited functional reserve [13], some surgeons still have concerns that they may actually be disadvantaged by the longer operative times usually described with this approach.

Previous studies of older patients undergoing laparoscopic colectomy, often defined as patients aged ≥ 70 years, have generally focused on short-term outcomes [14,15]. Our study aimed to identify factors associated with laparoscopic colectomy in older patients (aged ≥ 65 years) and to compare both short-term and long-term outcomes. We used the linked Surveillance, Epidemiology, and End Results (SEER)-Medicare database to permit comparisons of short-term outcomes such as length of stay, blood transfusions, intensive care monitoring, complication rates, and 30-day mortality, but also permit evaluation of long-term survival. A propensity score model incorporating patient factors including comorbidities and hospital characteristics was used to control for potential selection bias in assignment of surgical approach.

## Methods

### Patients and Data Sources

This study was approved by the National Cancer Institute (NCI), SEER, and University Hospitals Case Medical Center's Institutional Review Board. Informed consent was not obtained due to lack of patient identifiers. NCI's SEER tumor registry collects reliable patient-specific information from cancer registries [16]. Over the study period, SEER expanded to cover 25% of the U.S. population. Linking SEER to Medicare claims creates a joint database of Medicare beneficiaries (predominantly aged ≥ 65 years) who were diagnosed with cancer while living in SEER areas.

Patients aged ≥ 65 years with incident colon cancer diagnosed 1996--2002 presenting with localized, regional, or distant disease according to SEER who underwent colectomy as indicated by Medicare claims were included. Health Maintenance Organization enrollees and Medicare Part B non-participants from 12 months before to 3 months after diagnosis were excluded due to lack of claims data regarding outpatient and physician services [16]. Unstaged patients and those with carcinoma in situ, prior malignancy, familial adenomatous polyposis, or inflammatory bowel disease were excluded. Patients with transverse colon or rectal carcinoma were excluded as per prior RCTs [5,8,17].

### Measures

#### Patient and tumor characteristics

Demographic data (age, race, marital status, and gender), lymph node involvement, and stage at presentation were obtained from SEER. Comorbidities were assessed using a modified (malignancy and metastatic cancer excluded) version of the Deyo adaptation of the Charlson comorbidity index [18] based on International Classification of Diseases, 9th revision-clinical modification (ICD-9-CM) diagnosis codes from ≥ 1 inpatient (MedPAR) or ≥ 2 outpatient (Standard Analytical File) claims filed within a year to a month prior to cancer diagnosis [19]. Tumor site was dichotomized as left-sided (splenic flexure to sigmoid colon) or right-sided (cecum to hepatic flexure).

#### Colectomy

Colectomies occurring 1 month before to 6 months after cancer diagnosis were identified from MedPAR claims dated 1995-2003 using the following ICD-9-CM procedure codes: right hemicolectomy (45.73), left hemicolectomy (45.75), or sigmoidectomy (45.76). Colectomy claims including an ICD-9-CM procedure code for laparoscopy (54.21) with the same procedure date were considered laparoscopic [20-24]. Colectomy claims with ICD-9-CM diagnosis codes V64.4 or V64.41 (conversion of a laparoscopic procedure to open) were analyzed as laparoscopic colectomies similar to an intention-to-treat analysis. We could not distinguish laparoscopic from laparoscopic hand-assisted colectomies. Emergent cases, defined by admitting diagnoses of obstruction (560.89, 560.9), perforation (569.83), or peritonitis (567.0, 567.2x, 567.8x, 567.9), were included.

#### Hospital/Geographic Characteristics

Indirect medical education payment on a colectomy claim was considered evidence of performance at a teaching hospital [25,26]. Urban hospital and NCI cancer center designation were obtained from NCI's Hospital Files (1996, 1998, 2000-2003). U.S. Census 2000 data were obtained from the Patient Entitlement and Diagnosis Summary File regarding census tract median income, education level (% of adults with high school only education), and population density based on patient residence. Zip code data were imputed for missing census tract-level variables. SEER registries were grouped geographically into 5 regions: 1) West (San Francisco, Los Angeles, San Jose, Greater California, Seattle, Hawaii), 2) Mountain (Utah, New Mexico), 3) Midwest (Detroit, Iowa), 4) Northeast (Connecticut, New Jersey), and 5) South (Kentucky, Louisiana, Georgia).

#### Length of Stay, Transfusion Requirements, Intensive Care Monitoring, and Complications

MedPAR length of hospitalization, blood pints furnished, and intensive care indicator variables were used to determine length of hospitalization, transfusions, and intensive care monitoring, respectively. Colectomy claims were evaluated for complications using ICD-9-CM diagnosis codes for accidental laceration (998.2, E870.0), postoperative hemorrhage (998.1), posthemorrhagic anemia (285.1), anesthetic reaction (995.4, E938, E945.2), wound dehiscence (998.3), peritonitis (567.2), ileus (560.1), gastrointestinal hemorrhage (578, 578.0, 578.1, 578.9), gastrointestinal complications (997.4), intestinal fistula (569.81), stomach or duodenal fistula (537.4), and postoperative infection (998.5).

#### Chemotherapy Use

MedPAR, National Claims History, and Standard Analytical File claims were evaluated for chemotherapy administration occurring from 1 month prior to 6 months after cancer diagnosis using the following: chemotherapy-related ICD-9-CM procedure (99.25) and diagnosis codes (V58.1, V58.11, V67.2, V66.2); Healthcare Common Procedure Coding System codes for chemotherapy administration (Q0083-Q0085) and chemotherapeutic agents (J9000-J9999); and revenue center codes for chemotherapy (0331, 0332, 0335). As previously validated, chemotherapy use was imputed for claims consistent with chemotherapy administration or encounter where no drug was specified [27].

### Statistical Analysis and Definitions

Perioperative mortality was defined as death within 30 days of colectomy. Continuous covariates were compared using independent samples *t*-tests or Wilcoxon rank sum tests as appropriate. Categorical covariates were compared with chi-square tests. Unadjusted and adjusted logistic regression was performed to identify factors associated with laparoscopic colectomy. Data were analyzed with SAS (9.1 for Windows; SAS Institute Inc., Cary, NC).

#### Survival Analysis

Survival time was measured from colectomy to date of death. Survival times were administratively censored on December 31^st^, 2004. The Kaplan-Meier method was used to estimate survivor functions. Survival curves were compared with the log-rank test. We developed univariate and multivariate Cox proportional hazards models as well as a multivariate Cox proportional hazards. Covariates were assessed for time dependence to evaluate potential proportional hazards assumption violations.

#### Propensity Score Analysis

Because of nonrandom treatment allocation, a propensity score (PS) model was used to reduce bias resulting from differences in observed covariates between LC and OC groups. A propensity score is the conditional probability that a patient will be assigned to a particular treatment [28], in this case laparoscopic colectomy. To generate propensity scores, a non-parsimonious logistic regression model incorporating variables felt to be LC predictors and clinically plausible interaction terms was developed with laparoscopic colectomy as the dependent variable. LC patients were matched 1:1 to OC patients by propensity scores, resulting in 424 OC patients (designated the OC-Matched, or OC-M subset) with the same pre-intervention probability of undergoing LC as the 424 LC patients. Continuous outcomes were compared in the PS-matched groups using paired *t*-tests or Wilcoxon signed rank test as appropriate.

## Results

27,436 patients who met inclusion and exclusion criteria for incident colon cancer undergoing colectomy were identified from the SEER-Medicare database. Of these, 27,012 (98.5%) underwent open colectomy. Only 424 cases (1.5%) were identified as LC, of which 13.7% (n = 58) were converted to open. Mean age at cancer diagnosis was 77.9 years (standard deviation [SD] 7.1), reflecting an older population. Patients were predominantly female (58.3%, n = 16,006) and white (86.3%, n = 23,673). Blacks comprised 7.4% of the cohort (n = 2,020), and individuals of other/unknown ethnicity the remaining 6.4%. Only 21.5% (n = 5,889) of patients had a Charlson score ≥ 1. 41.6% of all cases presented with localized stage (n = 11,407), 44.0% with regional stage (n = 12,077), and 14.4% with distant stage (n = 3,952). 59.0% of colectomies occurred in cases diagnosed in 2000 or later, reflecting SEER's expansion in 2000. 50.1% of cases (n = 13,741) occurred in teaching hospitals.

Baseline characteristics of the cohort by surgical approach are shown (Table 1) with corresponding p-values. The groups were similar with respect to age, race, gender, marital status, comorbidities, and disability status. LC patients were less likely to have left-sided tumors (p = 0.001). The LC rate increased over the study period from 1.0% (30/2,908) of colectomies in 1996 to 1.8% (101/5,465) in 2002 (p = 0.001). LC cases were associated with areas with higher income, better education, higher population density, and SEER registries in the western U.S., especially California.

**Table 1:** Baseline Characteristics in Overall Cohort by Surgical Approach

Variable	LC (n = 424)	OC (n = 27,012)	P value^a^
Mean Age at Dx ± SD, years	77.5 ± 7.3	77.9 ± 7.1	0.034

Female (%)	234 (55.2)	15,772 (58.4)	0.185

Mean Charlson score ± SD	0.31 ± 0.83	0.38 ± 0.90	0.099

Disabled (%)	22 (5.2)	1,743 (6.5)	0.293

Weekend day admission (%)	67 (15.8)	4,334 (16.0)	0.893

**Race**			

White (%)	370 (87.3)	23,303 (86.3)	
	
African-American (%)	35 (8.3)	1,985 (7.4)	0.236
	
Other (%)	19 (4.5)	1,724(6.4)	

**Marital status**			

Married (%)	210 (49.5)	13,051 (48.3)	
	
Separated/Not married (%)	200 (47.2)	12,939 (47.9)	0.807
	
Missing (%)	14 (3.3)	1,022 (3.8)	

Emergent presentation (%)	12 (2.8)	1,272 (4.7)	0.069

Left-sided tumor (%)	125 (29.5)	10,016 (37.1)	0.001

Large metro area (%)	309 (72.9)	14,775 (54.7)	< 0.001^†^

**Data for patient's census tract**			

Income,^b ^$US	56,075 ± 27,809	50,024 ± 22,898	< 0.001

Education level^c ^± SD	23.0 ± 9.6	27.4 ± 10.0	< 0.001

Mean census tract density^d ^± SD	2,247 ± 3,298	1,768 ± 2,661	0.003

Census tract data imputed (%)	28 (6.6)	2,474 (9.2)	0.070

**Geographic region of SEER Registry**			

West (%)	223 (52.6)	9,286 (34.4)	
	
Mountain (%)	27 (6.4)	1,490 (5.5)	
	
Midwest (%)	44 (10.4)	6,807 (25.2)	< 0.001
	
Northeast (%)	75 (17.7)	5,789 (21.4)	
	
South (%)	55 (13.0)	3,640 (13.5)	

**Hospital Characteristics**			

Teaching hospital (%)	226 (53.3)	13,515 (50.0)	0.182

Urban hospital (%)	402 (94.8)	23,256 (86.1)	< 0.001

NCI Cancer Center (%)	24 (5.7)	728 (2.7)	< 0.001

^a ^P value from independent sample *t*-tests for continuous variables or chi-square tests for categorical variables

^b^Census tract median income

^c^Mean % of individuals with high school only education in the census tract

^d^In persons per square kilometer

OC cases took place in 1,135 of 1,136 hospitals in the cohort, compared with 174 hospitals for LC cases. LC cases were more common than OC cases in teaching hospitals (53.3% versus 50.0%) and were associated with urban hospitals and NCI-designated cancer centers, but not teaching hospitals. Accordingly, among hospitals at which LC cases were performed, 92.5% (n = 161) were urban and 4.6% (n = 8) were NCI-designated cancer centers, compared with 73.1% (n = 830) and 2.3% (n = 26), respectively, for hospitals at which OC cases were performed.

### Stage at Presentation, Nodal Status and Chemotherapy Receipt

LC patients presented with earlier stage than OC patients (p = 0.005). Localized disease patients comprised the largest proportion (46.9%) in the LC group, compared with regional disease patients (44.0%) in the OC group. Distant disease patients comprised 14.5% (n = 3,908) of the OC group, versus only 10.4% of LC patients (n = 44). The proportion of node-positive cases was similar between LC and OC groups (34.9% versus 38.8%, respectively; p = 0.24), as was receipt of chemotherapy within 6 months of diagnosis (27.6%, LC versus 28.0%, OC).

### Factors associated with Laparoscopic Colectomy

On univariate analysis, factors associated with laparoscopic colectomy included California residence, residence in a large metropolitan area, National Cancer Institute cancer center designation, urban hospital setting, localized stage, and residence in areas with higher census tract density and census tract median income. In addition, as the difference between the year of cancer diagnosis and the year 1996 increased, the associated odds ratio increased, reflecting the increase in the rate of laparoscopic colectomy over time. Left-sided tumors, regional or distant stage, and areas with lower census tract education were less likely to be associated with laparoscopic colectomy. On multivariate analysis, factors that remained associated with laparoscopic colectomy in this elderly population included California residence, residence in a large metropolitan area, and year of cancer diagnosis relative to 1996, while left-sided tumors, regional or distant stage, and areas with lower census tract education were less likely to be associated with laparoscopic colectomy (Table 2).

**Table 2:** Multivariate Analysis of Factors Associated with Laparoscopic Colectomy

Variable	Estimate	OR	95% CI	P value*
Year of diagnosis relative to 1996	0.0891	1.093	(1.039, 1.150)	0.001

Stage				

Regional (relative to localized)	-0.1583	0.854	(0.696, 1.047)	0.01°

Distant (relative to localized)	-0.4730	0.623	(0.448, 0.866)	

Left-sided tumor	-0.3260	0.722	(0.584, 0.892)	0.003

California residence	0.6293	1.876	(1.499, 2.349)	< 0.0001

Large metropolitan area	0.4975	1.645	(1.308, 2.068)	< 0.001

Education level^a^	-0.0221	0.978	(0.967, 0.990)	0.0002

NCI cancer center designation	0.8637	2.372	(1.555, 3.619)	< 0.001

^a^Mean % of individuals with high school only education in the census tract

### Short-Term Outcomes

Median length of stay in the LC group was 7 days (mean 8.3 days, SD 6.2). Median length of stay in the OC group was 8 days (mean 10.6 days, SD 7.6). The mean number of blood pints furnished did not differ between LC and OC groups (LC 0.13, OC 0.24). Among LC cases, 25.2% (n = 107) had intensive care unit (ICU) monitoring during the hospitalization, compared with 31.5% (n = 8,517) in the OC group (p = 0.006). OC patients had a higher complication rate than LC patients (26.3% versus 21.5%, p = 0.03). Rates of ileus or gastrointestinal complications, the most common complications, did not differ between the LC and OC groups. The overall OC group had a significantly higher rate of postoperative hemorrhage or posthemorrhagic anemia than the LC group (9.9% versus 5.7%, p = 0.003). 30-day mortality was 3.3% (n = 14) in the LC group, significantly lower than the overall OC group rate of 5.8% (n = 1,565; p = 0.03).

### Long-Term Survival

**Figure 1:**
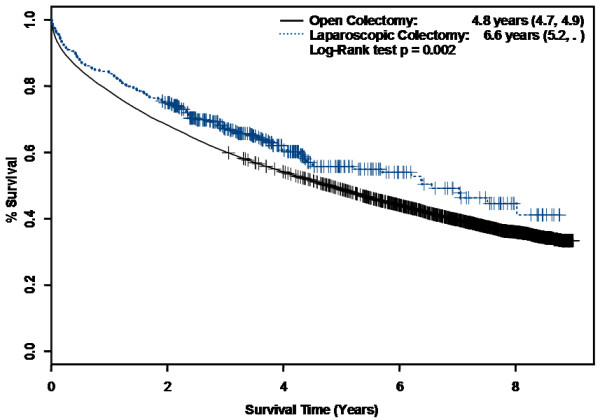
**Kaplan-Meier Survival Curves by Surgical Approach**.

Median survival was 6.6 years in the LC group (95% CI 5.2, xxx.) and 4.8 years in the overall OC group (95% CI 4.6, 4.9). Two-year, three-year, and 5-year survival rates were better in the LC than the OC group (75.0% versus 68.3%; 67.0% versus 60.2%; and 55.8% versus 48.9%, respectively). The log-rank test comparing long-term survival curves stratified by surgical group (Figure 1) was statistically significant (p = 0.002).

Demographic, tumor, hospital, and geographic characteristics in the overall cohort were next examined in univariate analysis for significant differences in time to death (Table 3). Laparoscopic colectomy was associated with improved survival compared with open colectomy (HR 0.79, 95% CI 0.68, 0.92). However, after adjusting for comorbidities, demographic characteristics, and tumor characteristics, LC was no longer associated with improved survival compared with OC. The final multivariate Cox proportional hazards model incorporating interaction terms and time-dependent covariates is shown (Table 4).

**Table 3:** Univariate Predictors of Long-Term Survival in Overall Cohort

Variable	Hazard Ratio	95% CI	P value^a^
Laparoscopic Colectomy	0.787	0.677-0.916	0.0012

Female Gender	0.936	0.905-0.968	0.0002

Black Race	1.204	1.132-1.280	< 0.0001^b^
	
Other Race	0.886	0.825-0.952	

Emergent Presentation	1.874	1.751-2.006	< 0.0001

Weekend Admission	1.120	1.070-1.173	< 0.0001

Large Metro Area	1.085	1.049-1.123	< 0.0001

Left-sided Tumor	0.935	0.903-0.968	0.0001

California Registry	1.005	0.967-1.044	0.8065

Disabled	1.268	1.189-1.352	< 0.0001

Positive Nodes	2.337	2.259-2.417	< 0.0001

Teaching Hospital	0.981	0.948-1.014	0.2564

Separated/Not Married	1.385	1.338-1.433	< 0.0001^c^
	
Marital Status Unknown	1.171	1.069-1.283	

Chemotherapy Receipt	0.923	0.889-0.959	< 0.0001

Regional Stage Disease	1.686	1.620-1.755	< 0.0001^d^
	
Distant Stage Disease	6.33	6.043-6.637	

Urban Hospital	1.013	0.964-1.064	0.6085

NCI Cancer Center	0.854	0.767-0.950	0.0028

Age at dx (1-year change)	1.050	1.048-1.053	< 0.0001

Year of dx relative to 1996	0.995	0.986-1.004	0.3220

Census tract median income ($1,000 change)	0.997	0.997-0.998	< 0.0001

Census tract % high school only education	1.003	1.001-1.004	0.0012

Census tract density (1,000 persons/unit change)	1.005	1.003-1.007	< 0.0001

Charlson index (1-unit change)	1.244	1.225-1.264	< 0.0001

Dx, Diagnosis.

^a ^Partial likelihood ratio test.

^b ^Compared with white race.

^c ^Compared with married.

^d ^Compared with localized stage.

**Table 4:** Final Cox Proportional Hazards Model in Overall Cohort

Variable	Hazard Ratio	95% CI	P value^a^
Laparoscopic Colectomy	0.902	0.776-1.050	0.1838

Female Gender	0.846	0.818-0.876	< 0.0001

Black Race	1.174	1.104-1.249	< 0.0001^b^

Emergent Presentation	1.419	1.325-1.519	< 0.0001

Positive Nodes	1.897	1.812-1.986	< 0.0001

Chemotherapy Receipt	3.380	2.040-5.600	< 0.0001

Chemotherapy*ln(survival time)	1.356	1.314-1.399	< 0.0001

Regional Stage	1.264	1.201-1.329	< 0.0001^c^

Distant Stage	5.560	5.236-5.903	< 0.0001^c^

Regional Stage*ln(survival time)	0.967	0.941-0.993	0.0127

Distant Stage*ln(survival time)	1.037	1.006-1.068	0.0184

Age at Dx (1-year change)	1.056	1.053-1.059	< 0.0001

Age at Dx*ln(survival time)	1.003	1.001-1.004	0.0011

Age at Dx*Chemotherapy	0.978	0.972-0.985	< 0.0001

Charlson index (1-unit change)	1.257	1.237-1.277	< 0.0001

Dx, Diagnosis.

^a ^Wald test.

^b ^Compared with white/other race.

^c ^Compared with localized stage.

### Propensity Score Analysis

A non-parsimonious multivariable logistic propensity model was created incorporating all observed covariates potentially associated with LC that would have been known prior to decision-making regarding surgical approach. Through this model propensity scores reflecting the probability of undergoing LC were generated. LC patients were then matched 1:1 to OC patients on the basis of the propensity score, and the matched cohort was re-analyzed. After PS matching, mean length of stay remained significantly longer with the OC-Matched (OC-M) subset (10 days, SD 8.9) compared with the LC group (p < 0.001). As before, blood pints furnished did not differ after PS matching (LC 0.13, OC-M 0.17). ICU Monitoring remained more common in the OC-M group after PS matching (OC-M, 33.7% versus LC, 25.2%). After PS matching, complication rates were no longer different between the two groups (OC-M, 26.7% versus LC, 21.5%, p = 0.09). Likewise, the rate of postoperative hemorrhage or posthemorrhagic anemia was no longer significantly different between groups (OC-M, 9.0% versus LC, 5.7%). Differences in 30-day mortality disappeared after PS matching (OC-M, 4.3% [n = 18]).

**Figure 2:**
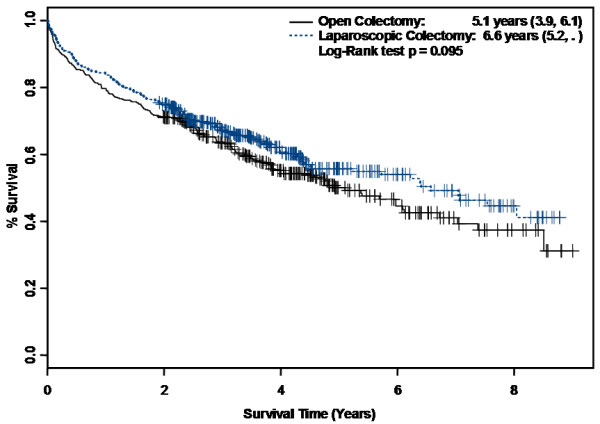
**Kaplan-Meier Survival Curves by Surgical Approach After Propensity Score Matching**.

Median survival improved from 4.8 years in the overall OC group to 5.1 years (95% CI 3.9, 6.1) in the OC-M subset. Likewise, 2-year, 3-year, and 5-year survival in the OC-M subset were also improved compared to the overall OC group (2-year survival, 71.2% versus 68.3%; 3-year survival, 63.4% versus 60.2%; 5-year survival, 50.0% versus 48.9%). Kaplan-Meier survival curves stratifying survival by surgical treatment in the PS matched cohort are shown (Figure 2). The log-rank test was not statistically significant (p = 0.095). Table 5 displays the final Cox proportional hazards model including interactions and time-dependent variables. As with the multivariate Cox proportional hazards model in the overall cohort, laparoscopic colectomy was not associated with improved survival (HR 0.94, 95% CI 0.76, 1.15).

**Table 5:** Final Cox Proportional Hazards Model in Propensity Score-Matched Cohort

Variable	Hazard Ratio	95% CI	P value^a^
Laparoscopic Colectomy	0.936	0.760-1.152	0.5301

Female Gender	0.996	0.805-1.232	0.9693

Positive Nodes	2.028	1.504-2.735	< 0.0001

Chemotherapy Receipt	0.550	0.423-0.717	< 0.0001

Chemotherapy*ln(survival time)	1.460	1.222-1.744	< 0.0001

Regional Stage disease	1.520	1.103-2.095	0.0105^b^

Distant Stage disease	6.463	4.409-9.472	< 0.0001^b^

^a ^Wald test.

^b ^Compared with localized disease.

## Discussion

This project identified factors associated with laparoscopic colectomy in an older population and compared short-term and long-term outcomes. The rate of LC in the overall cohort was low at 1.5%, potentially due to the perceived morbidity of performing LC in elderly patients. Population-based studies using the National Cancer Data Base and the Nationwide Inpatient Sample over a similar time period found LC rates of 4.6% and 3.3%, respectively [20,29]. However, these studies included patients aged < 65, and the Nationwide Inpatient Sample study relied on a cancer diagnosis code for the hospitalization rather than cancer registry data. Notably, in the current study, LC cases were more likely to occur at hospitals with NCI cancer center designation or in individuals residing in areas with higher education levels or larger populations (Table 2). These factors probably reflect tertiary care practice patterns.

Consistent with RCT results [2,5], LC was associated with shorter hospitalizations. Relatively high ICU monitoring rates were seen in the two treatment groups, with higher rates in the OC and OC-M groups (31.5% and 33.7%, respectively) than LC (25.2%). Direct comparison with RCTs is not possible due to lack of corresponding RCT data. Nonetheless, these results are intriguing given the older population in this study. Although a laparoscopic approach theoretically may be associated with a higher risk of cardiopulmonary complications (and hence more intensive monitoring) due adverse effects of pneumoperitoneum on the cardiovascular system, our analysis does not support such a hypothesis.

Complication rates were relatively low except for bleeding, ileus, and gastrointestinal complications. In particular, the postoperative infection rate was 0.2% in the OC group, with no cases in the LC group. The wound infection rate in RCTs has ranged between 3%-12% [2,30]. Postoperative infection was likely undercoded in this study, and discharged patients that were readmitted with infection were not captured. Although several RCTs have demonstrated decreased blood transfusion requirements with LC [2,6,30], in the current study, transfusion requirements did not differ by surgical group. 30-day mortality rates (ranging from 3.3% in the LC group to 5.8% in the overall OC group) in our study were similar to the rate of 5.6% previously reported in an analysis by Rabeneck et al. of Veterans Affairs patients aged ≥ 65 years undergoing colon cancer resection [31].

The 5-year survival rates in our study ranged between a low of 48.9% for the overall OC group to 55.8% for the LC group. These rates are much lower than the 5-year overall survival rates of 74.6%-76.4% reported from the COST study, a randomized controlled trial comparing laparoscopically assisted colectomy to open colectomy [7]. The median age of patients in the COST study was younger than that in the current study [5]. The lower 5-year survival in the current study probably reflects increased mortality and comorbidities in this older population and the fact that this series was not a selected group of patients for a randomized controlled trial. Consistent with our results, the Rabeneck study reported a 5-year overall survival rate of 46.6% in patients aged ≥ 65 years versus 57.6% in patients aged < 65 years following resection for colon cancer [31].

Long-term survival appeared more favorable in the LC group than the overall OC group on Kaplan-Meier analysis (Figure 2). These results were likely partially attributable to confounding by stage and nodal status, both of which favored the LC group. An adjusted survival analysis using Cox proportional hazards models in the overall cohort revealed no survival difference. Similarly, in the PS matched cohort, long-term survival did not differ by surgical approach. Our findings are therefore consistent with most previous RCTs. In contrast, a prior population-based study found higher 5-year survival rates with laparoscopic-assisted colectomy (64.1%) compared to open colectomy (58.5%) [29]. However, those results were not adjusted for comorbidities which may preferentially affect older individuals. Our results suggest that a laparoscopic approach does not seem to impact long-term survival compared with an open approach in older patients.

Our study had several strengths that should be noted. Its focus on older individuals is particularly relevant due to the aging population and the predominance of colorectal cancer in this age range. This study analyzed factors associated with the use of laparoscopic colectomy for colon cancer in older individuals; previous studies of laparoscopic colectomy in older individuals have often been limited to single centers. The SEER-Medicare dataset provided information on individuals from a variety of areas across the United States and allowed identification of comorbid conditions diagnosed prior to cancer diagnosis which could have affected survival.

This study nonetheless had several limitations. First, Medicare claims were used to identify LC cases because SEER does not differentiate laparoscopic from open surgical approach. This approach may have led to LC underdetection. Of note, a recent analysis of SEER-Medicare data including cases diagnosed through 2005 evaluating outcomes after colorectal cancer resection found that laparoscopic cases comprised < 2% of cases [32]. Second, the unvalidated blood pints furnished variable may be undercoded and might not correlate with ICD-9-CM blood transfusion procedure codes [33]. We also used procedure codes to measure ICU monitoring but these codes have not been well validated. Third, use of the combination of ICD-9-CM procedure codes for colectomy and laparoscopy on the same date to indicate laparoscopic colectomy has not been validated, although similar approaches have been utilized in other studies [20-24]. Fourth, except for incorporation of hospital characteristics, this analysis did not account for clustering of LC cases in a subset of hospitals.

## Conclusions

In this analysis of older individuals with colon cancer, laparoscopic colectomy was associated with factors that probably correlate with tertiary care practice patterns. Laparoscopic colectomy was associated with shorter hospitalizations and less ICU monitoring. Although 5-year survival was lower than that reported in RCTs, laparoscopic colectomy was not associated with worse survival compared with open colectomy. Laparoscopic colectomy appears to be a reasonable option in older patients with colon cancer which reduces hospital stay and intensive care use.

## List of abbreviations

LC: laparoscopic colectomy; OC: open colectomy; RCT: randomized controlled trial; SEER: Surveillance, Epidemiology, and End Results; NCI: National Cancer Institute; ICD-9-CM: International Classification of Diseases, 9th revision-clinical modification; PS: propensity score; OC-M: open colectomy-matched; SD: standard deviation; ICU: intensive care unit.

## Competing interests

The authors declare that they have no competing interests.

## Authors' contributions

LCC conceived of the study, participated in the study design, performed the analysis, interpreted the results, and wrote the manuscript. CPD participated in study design, interpretation of results, and editing the manuscript. GSC participated in study design, acquisition of data, interpretation, and editing the manuscript. All authors read and approved the final manuscript.
